# The SCOPE framework – implementing the ideals of responsible research assessment

**DOI:** 10.12688/f1000research.140810.1

**Published:** 2023-09-28

**Authors:** Laura Himanen, Erica Conte, Marianne Gauffriau, Tanja Strøm, Baron Wolf, Elizabeth Gadd

**Affiliations:** 1 CSC – IT Center for Science, Keilaranta 14, Espoo, 02101, Finland; 2 Unity Health Toronto, Toronto, Ontario, L1Z 1P3, Canada; 3 IT University of Copenhagen, Rued Langgaards vej 7, Copenhagen, DK-2300, Denmark; 4 Oslo Metropolitan University, Olso, 1383, Norway; 5 University of Kentucky, Lexington, Kentucky, 40502, USA; 6 Loughborough University, Loughborough, England, LE11 3TU, UK

**Keywords:** Research evaluation, responsible research assessment, evaluation framework, SCOPE

## Abstract

**Background:** Research and researchers are heavily evaluated, and over the past decade it has become apparent that the consequences of evaluating the research enterprise and particularly individual researchers are considerable. This has resulted in the publishing of several guidelines and principles to support moving towards more responsible research assessment (RRA). To ensure that research evaluation is meaningful, responsible, and effective the International Network of Research Management Societies (INORMS) Research Evaluation Group created the SCOPE framework enabling evaluators to deliver on existing principles of RRA. SCOPE bridges the gap between principles and their implementation by providing a structured five-stage framework by which evaluations can be designed and implemented, as well as evaluated.

**Methods:** SCOPE is a step-by-step process designed to help plan, design, and conduct research evaluations as well as check effectiveness of existing evaluations. In this article, four case studies are presented to show how SCOPE has been used in practice to provide value-based research evaluation.

**Results:** This article situates SCOPE within the international work towards more meaningful and robust research evaluation practices and shows through the four case studies how it can be used by different organisations to develop evaluations at different levels of granularity and in different settings.

**Conclusions:** The article demonstrates that the SCOPE framework is rooted firmly in the existing literature. In addition, it is argued that it does not simply translate existing principles of RRA into practice, but provides additional considerations not always addressed in existing RRA principles and practices thus playing a specific role in the delivery of RRA. Furthermore, the use cases show the value of SCOPE across a range of settings, including different institutional types, sizes, and missions.

## 1 Introduction

This article introduces the SCOPE framework for responsible research assessment (RRA) developed by the International Network of Research Management Societies’ (INORMS) Research Evaluation Group (REG) and situates it within the sector-wide drive towards more meaningful and robust research evaluation practices. It begins with a short overview of RRA and some of the underpinning declarations and principles that form its foundation. The need for the SCOPE framework as a mechanism which both delivers, and expands upon, those principles is then outlined. The SCOPE framework is then described and justified with reference to the scholarly literature. Four use cases demonstrating the wide applicability of SCOPE are then provided and some conclusions regarding both its usefulness and its applicability are drawn, highlighting both its strengths and weaknesses.

## 2 Responsible research assessment

Research and researchers are heavily evaluated. There are many reasons for this. First and foremost, evaluation is a distinctive characteristic of science itself, as the demand for verifiability of results puts scientific research under the exacting scrutiny of fellow experts. As Robert Merton puts it ( Merton, 1973, p. 276), “the activities of scientists are subject to rigorous policing, to a degree perhaps unparalleled in any other field of activity”. Evaluation has a major role in the knowledge-making process as both a gatekeeper and a legitimator of knowledge, and as a process itself: evaluation sets standards of research quality ( Lamont & Huutoniemi, 2011). Demands for greater accountability, linked with diminishing funding, have also created expectations for universities to be both efficient and accountable. As such evaluation is essential to ensure that funding is used in the best possible way ( Geuna & Martin, 2003). Research evaluation can also be seen as a governance tool to improve the quality of scholarship ( Gläser & Laudel, 2007). It is used for monitoring the activities of researchers, projects, programmes, departments, and institutions – even nations.

According to Geuna & Martin research evaluations tend to focus on four typical output measures: volume, quality, impact, and utility, with peer review and bibliometric measures as their main methods ( Geuna & Martin, 2003). While peer review is considered as the key evaluation mechanism of scholarly work ( Birukou *et al*., 2011), over the last two decades we have witnessed a strong increase in the use of quantitative techniques ( Van Leeuwen, 2004). As stated in the Leiden Manifesto ( Hicks *et al*., 2015, p. 431), “research metrics can provide crucial information that would be difficult to gather or understand by means of individual expertise.” Thelwall *et al*. argue that certain dimensions of scientific quality, such as scientific and societal impact and visibility, can be well-served by indicators ( Thelwall *et al*., 2015). However, others have argued that indicators used in researcher assessment do not capture the elements that reflect high-quality research and knowledge advancement ( Aubert Bonn & Pinxten, 2021b).

Whatever method is used, it has become apparent that the consequences, both intended and unintended, of evaluating the research enterprise and particularly individual researchers are considerable. Some of the negative consequences include, but are not limited to, the stress and burden on researchers and administrators, tension between colleagues, biased and unfair evaluations, and misalignment with organisational missions and values (see, e.g., Aubert Bonn *et al*., 2022; Aubert Bonn & Pinxten, 2021a; Benedictus *et al*., 2016; Gadd, 2021; Lebel & McLean, 2018; Moher *et al*., 2018; Morrish, 2017; Muller, 2018; Saenen *et al*., 2019; Wellcome Trust, 2020). As a response, within the last decade, several guidelines and principles have been published to support moving towards more responsible modes of research evaluation, now commonly referred to as RRA.

## 3 Key RRA principles and guidelines

In 2012, during the Annual Meeting of The American Society for Cell Biology in San Francisco, a group of editors and publishers of scholarly journals developed a set of recommendations known as the San Francisco Declaration on Research Assessment (DORA) ( *DORA*, 2012). Since then, it has become a worldwide initiative, with over 23,000 individual signers and close to 2,900 organizational signers as of June 2023. DORA consists of 18 recommendations that are aimed at funding agencies, academic institutions, journals, organizations that supply metrics, and individual researchers. Even though it highlights the need to consider the value and impact of all research outputs in assessment, the recommendations focus primarily on practices relating to peer reviewed journal articles as the central mechanism by which research is currently assessed. A major recommendation is to eliminate the use of journal-based metrics and to assess research on its own merits rather than on the basis of the journal in which it is published. It also recommends the need to use a broad range of impact measures including qualitative indicators. For publishers and researchers, the recommendations also encompass responsible authorship practices, and for publishers and organizations that supply metrics related to issues of openness and transparency be included.

In 2015, the Leiden Manifesto for research metrics was published providing ten principles to guide bibliometric-based evaluation ( Hicks *et al*., 2015). The authors of the manifesto were alarmed by the pervasive misapplication of indicators to the evaluation of scientific performance, and as a response presented the ten principles, “so that researchers can hold evaluators to account, and evaluators can hold their indicators to account” (ibid., 430). The first three principles consider the role of metrics in research evaluation on a general level. They remind us that the role of quantitative metrics in research assessment should be to support qualitative, expert assessment. Indicators should not be allowed to substitute informed judgement. When indicators are used, they should consider diverse research missions, and measure performance against those missions. Also, using metrics in research assessment poses a risk to locally relevant research as in many parts of the world research excellence is equated with English-language publications. The remaining seven principles are more practical in nature, considering issues around data collection and analytical processes, the use of indicators and the effects they have on the system.

Shortly after the Leiden Manifesto was produced, an independent review of the role of metrics in research assessment and management was published called The Metric Tide ( Wilsdon *et al*., 2015). The review examined the role of metrics in the UK Research Excellence Framework (REF) conducted in 2014, but it also explored wider issues by looking at the applicability of metrics within different research systems, comparing the peer review system with metric-based alternatives, as well as examining the effects of the growing use of quantitative indicators on different aspects of research culture.

As part of the review, building on the concept of ‘responsible research and innovation’ the authors propose the notion of ‘responsible metrics’ as a way of framing appropriate uses of quantitative indicators in, inter alia, the assessment of research. Their understanding of responsible metrics is built on five principles that have some commonality with elements of DORA and the Leiden Manifesto. They call for recognition that quantitative evaluation should support expert assessment, for basing metrics on the best possible data in terms of accuracy and scope and keeping data collection and analytical processes open and transparent. Accounting for variation by field and using a range of indicators is recommended, as well as recognizing and anticipating the systemic and potential effects of indicators.

The Hong Kong Principles for assessing researchers: Fostering research integrity was published in 2020 ( Moher *et al*., 2020). The starting point for the Hong Kong Principles differed from the three sets of principles previously described, in that its focus is on the need to recognize and reward researchers for behaviours that strengthen research integrity. The authors state that for knowledge to benefit research and society, it must be trustworthy, robust, rigorous, and transparent. The five principles call for researchers to be assessed on accurate and transparent research reporting and engaging with open science practices. In line with DORA, the Leiden Manifesto and the Metric Tide, the Hong Kong Principles call for valuing a broad range of research and scholarship, such as replication, innovation and translation, and other contributions to responsible research and scholarly activity, such as peer review activity, mentoring and outreach.

The most recent addition to the responsible research assessment landscape is the Agreement on Reforming Research Assessment which encompasses many of the ambitions of earlier declarations and expands upon them by requiring institutions to commit to actually changing their practice within an agreed timeframe ( European University Association *et al*., 2022). The Agreement was published in 2022, and more than 350 organisations from over 40 countries were involved in the drafting. Signing up to the agreement became possible in September 2022 and by July 2023 almost 600 organisations had signed. The Agreement sets a shared direction for changes in assessment practices, as well as a timeframe for implementing reforms. In signing, organisations make four core commitments: to recognize a broader diversity of outputs, practices and activities when assessing research; to base assessment primarily on qualitative judgement supported by quantitative indicators where appropriate; to avoid inappropriate uses of journal and publication metrics and to avoid using university rankings in researcher assessment.

## 4 The SCOPE Framework and RRA principles

In 2001 the International Network of Research Management Societies’ (INORMS) was formed to bring together research management societies and associations from across the globe. In recognition of the fact that research assessment was having a growing influence on the research management profession, INORMS established a Research Evaluation Group (INORMS REG) in 2018 to consider how best to ensure that research evaluation is meaningful, responsible, and effective. As part of the INORMS REG’s aim of guiding university leaders and practitioners in the adoption and practice of responsible research evaluation, they developed a framework that both enabled evaluators to deliver on existing principles of responsible assessment and to address some additional critical elements. As such, the SCOPE framework is a practical, five-stage step-by-step process for evaluating responsibly, supported by three overarching principles. Table 1 outlines how SCOPE seeks to deliver on some of the key elements of existing initiatives.

**Table 1.** The relationship between SCOPE and RRA principles.

Principle/Declaration	Objectives	Relationship with SCOPE
**San Francisco Declaration on Research Assessment (DORA), 2012** Seeks improved assessment of researchers and a better scholarly communication ecosystem.	18 recommendations for different stakeholders. The key themes are to: · Eliminate journal-based metrics. · Assess research on its own merits. · Take advantage of online publication possibilities.	SCOPE shares DORA’s vision for better researcher assessment & eliminating the poor use of journal metrics, but is broader in focus, overseeing the responsible assessment of any entity.
**Leiden Manifesto, 2015** Seeks more responsible use of bibliometrics in research assessment.	10 principles for the responsible use of bibliometrics in research assessment focused on: · Metrics supporting rather than supplanting expert assessment. · Mission-based performance assessment. · Accounting for variation by field in citation metrics.	SCOPE shares the Leiden Manifesto’s vision for contextualized use of bibliometrics, but is not limited to quantitative indicators, as it accounts for qualitative measures too.
**The Metric Tide, 2015** Seeks to guide a broad range of research assessment approaches.	Five principles for all forms of research assessment: · Robustness · Humility · Transparency · Diversity · Reflexivity	SCOPE also has a broad focus, but does not stop at providing principles, as it also provides a pragmatic, step-by-step process for evaluating responsibly that includes characteristics like value-led beginnings and a sense-checking probe stage.
**Hong Kong Principles, 2020** Seeks to reward practices that lead to researcher integrity rather than unhelpful & limited publication-based rewards.	Series of principles for assessing researchers that reward research integrity focused on: 1. Assessing responsible research practices 2. Valuing complete reporting 3. Rewarding the practice of open science 4. Acknowledging a broad range of research activities	SCOPE also offers value-based assessments, but does not prescribe what those values should be, instead letting the evaluators (together with the evaluated) generate the values that are most meaningful to them.

SCOPE bridges the gap between principles and their implementation by providing a structured and orderly framework by which evaluations can be designed and implemented as well as evaluated. Existing principles focus mainly on either evaluating a specific entity, like researchers in the case of DORA and Hong Kong Principles, or via a particular mechanism, like research metrics in the case of Leiden Manifesto and Metric Tide. SCOPE seeks to be applicable across the whole research ecosystem, enabling a responsible approach to evaluating any entity via any relevant mechanism.

As well as enabling the implementation of existing RRA principles, SCOPE also brings additional elements perceived to be necessary in the implementation of responsible assessments. These include three essential principles: 1) to evaluate with the evaluated; 2) to evaluate only where necessary; and 3) to evaluate with the appropriate expertise. It also addresses the need to be context-sensitive, to consider both qualitative and quantitative options, and to probe for unintended consequences and to evaluate your evaluation. A full 19-page guide to SCOPE is available on the INORMS REG webpage ( International Network Of Research Management Societies-Research Evaluation Group, 2021) and it is not our purpose to reproduce that here. However, in the next section we provide an outline of the SCOPE framework and principles in some detail situating it within the existing literature.

## 5 The principles of SCOPE

### 5.1 Evaluate only where necessary

**Figure 1.**
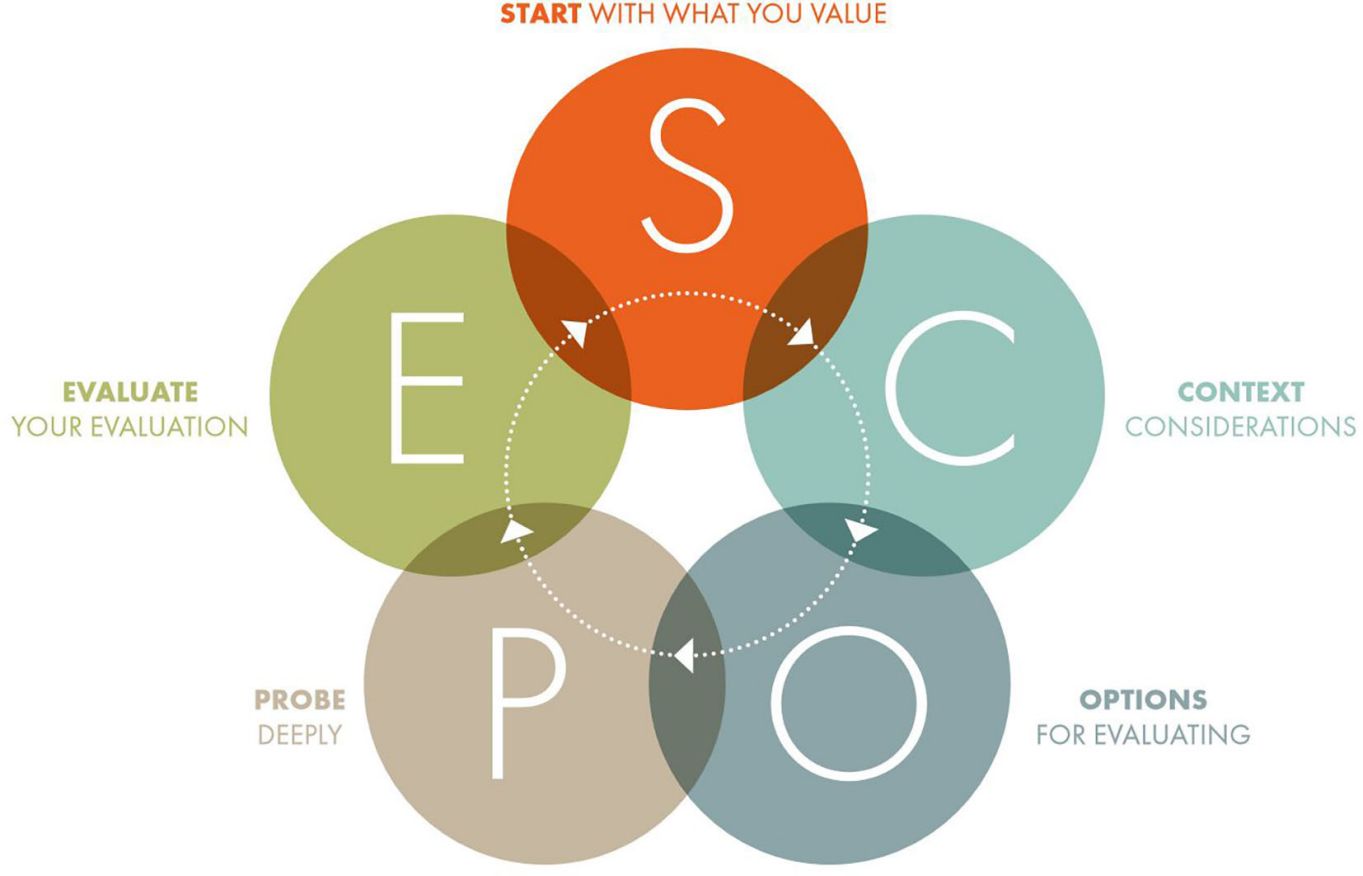
The SCOPE Framework (from The SCOPE Framework: a five-stage process for evaluating responsibly: https://doi.org/10.26188/21919527.v1).

The five stages of SCOPE presented in Figure 1 operate under three main principles. The first is to evaluate only where necessary. Hallonsten argues that science has been enormously productive even in times when quantitative performance evaluation was not a tool for science policy or university governance, that is, for most of modernity ( Hallonsten, 2021). He goes on to conclude that whilst the continuous evaluation of quality is an essential feature of the scientific knowledge production process, the same does not apply to the evaluation of ‘excellence’ and ‘relevance’ for the sake of increasing efficiency and accountability (ibid., 19-20).

Despite this, it is generally accepted that the Higher Education sector is now subject to neoliberal managerial approaches whereby if you can’t count it, it doesn’t count ( Feldman & Sandoval, 2018). This increased focus on evaluation to both monitor progress and to incentivise behavioural change has led to a significant increase in the volume of assessments to which researchers, groups and institutions are subject. Whilst over-evaluation is particularly problematic when the range of contributions being assessed is narrow (e.g., the volume, location and citedness of publications ( Saenen *et al*., 2019)) it remains problematic even when applied to a broader diversity of contributions. A new focus on open research practices, integrity and collegiality in our assessments doesn’t displace or even dilute a focus on publications and grant income, but simply expands the number of dimensions on which researchers are assessed. Many of these new dimensions are also not yet mature enough to be evaluated in a robust way at all levels of granularity, which can lead to well-intended but poorly designed evaluations based on limited data.

Poor evaluation design and an overfocus on evaluation for evaluation’s sake has been highlighted as one of the key drivers of many mental health issues in the sector, driving many researchers to seek posts in industry ( Gewin, 2022) or worse, to take catastrophic action ( Parr, 2014). In response to these considerations, the SCOPE framework urges evaluators to ask at the outset whether they need to evaluate at all, or whether an alternative approach might be taken (such as *enabling* open research practices rather than *evaluating* them). Where an evaluation is deemed necessary, the extent of the evaluation effort should be commensurate with the potential impact of the evaluation. For example, surveys should ask the minimum viable number of questions and the frequency of assessments should be considered carefully.

### 5.2 Evaluate with the evaluated

The second principle of SCOPE is to evaluate with the evaluated. The principles of co-design are now central to many domains including product and service design, policy design and of course research design itself ( Blomkamp, 2018; Moser, 2016; Steen *et al*., 2011). Co-design has also been a particularly important tool in efforts to meet equity, diversity and inclusion ambitions ( KPMG, 2022). The benefits of co-design are seen to be a more creative process, better outcomes, and greater buy-in by stakeholder communities. It would seem entirely appropriate then, especially given sector concerns about the volume, quality, and format of research assessments, that a principle of co-design and co-evaluation should be adopted.

In this vein, the Leiden Centre for Science and Technology Studies (CWTS) recently proposed ‘evaluative inquiry’ as a more enabling approach to providing material for assessment ( Fochler & De Rijcke, 2017). The approach seeks to present research work in ways that represent the complexity of actual practice by engaging with those practitioners instead of taking reductionist approaches to assessment for the sake of standardization (ibid., 34).

The use of SCOPE workshops to develop research evaluations are a good way of ensuring this principle is adhered to.

### 5.3 Draw on evaluation expertise

The third principle of SCOPE is to draw on evaluation expertise. The ready availability of bibliometric data and tools has led to concern from scientometricians around the rise of ‘armchair bibliometrics’ or ‘citizen scientometricians’ ( Leydesdorff *et al*., 2016). It is common to find academics in every discipline running bibliometric analyses to better understand research activity in their field. This has led others to plead ‘epistemic trespass’. Ballantyne defines epistemic trespassers as “thinkers who have competence or expertise to make good judgements in one field but move to another field where they lack competence – and pass judgement nevertheless” ( Ballantyne, 2019, p. 367). When it comes to research assessment, the fact that all researchers are regularly involved in assessing research proposals and applicants for research positions may give them greater confidence that they can expand this knowledge to designing research assessments. More recent emphasis on the need for responsible approaches to research assessment have brought into greater relief how easy it is to get research assessment design wrong. Clearly, the same rigour that is expected of academic research should be expected also of all evaluations of academic research.

## 6 The five-stage SCOPE framework

### 6.1 Start with what you value

The first stage of SCOPE, ***start with what you value***, is a critically important first step in any evaluation. It is about exploring what is valued about the particular entity being evaluated: putting the ‘value’ in e-‘valu’-ation. This approach resonates with the Humane MetricsHSS initiative which supports values-enacted frameworks for evaluating all aspects of scholarly activity, as well as with Leiden Manifesto’s second principle urging that the performance of institutions, groups or researchers should be measured against their missions ( Hicks *et al*., 2015).

An important question when considering what might be valued about an entity under evaluation, is to ask to *whom* the entity offers some value. For example, in a national university research assessment programme there are many stakeholders that all may value different things about the universities being evaluated: the treasury funding the outcomes, the government agency running the evaluation, the institutions themselves, the researchers who work in them, and so on. In line with the SCOPE principle of ‘evaluating with the evaluated’, the SCOPE approach would be to explore the question across a range of stakeholder perspectives and to seek to find consensus where possible.

If assessments are not developed in line with what stakeholders value about the entity being evaluated, too often they are made in line with what third parties value, or with historical values, or simply in accordance with the data we have available.

The problematic effects of relying on third party values such as the university rankings (see, e.g., Gadd, 2020, 2021; Van Raan, 2005) or national evaluation systems (see, e.g., Aagaard, 2015; Butler, 2005; Watermeyer *et al*., 2023) are well-documented. The key concern is that given Campbell’s Law (what we measure is what we get) ( Campbell, 1979), by ‘outsourcing’ our values to others, evaluators run the risk of producing scholarship and research practices that are not in line with their own mission or ambitions.

The practice of starting with the data that is easily available, and evaluating what can be measured rather than what is valued, is often criticised ( Lane *et al*., 2014). A common focus of such concerns is the over-use of bibliometrics in researcher recruitment and career assessment ( Saenen *et al*., 2021). Proponents of value-led assessment approaches argue that evaluations should not be reduced to the concept of measurable achievements only, as there are multiple contributions that research and researchers make both to scholarship and society ( Agate *et al*., 2020; Holtrop *et al*., 2020a).

At this stage of SCOPE it is important to maintain the first principle of ‘evaluating only where necessary’. Evaluators can fall into the trap of not taking the time to consider what is of the most value and therefore evaluate everything possible.

### 6.2 Consider the context

Discussions around what constitutes a ‘good’ or ‘bad’ indicator are abundant in the responsible research evaluation literature (e.g., Rijcke *et al*., 2016). However, whether an indicator (or indeed any assessment approach) can be determined as suitable or unsuitable depends on the context for that evaluation: what is being measured (entity and discipline) and for what purpose. For this reason, the second stage of SCOPE invites evaluators to ***consider the context*** of the evaluation.

Entities under evaluation can range from nations through to individuals, and on each level different types of consideration need to be addressed. This is especially the case when those entities are seen through different disciplinary lenses ( Holtrop *et al*., 2020b; Konkiel, 2018; Puuska, 2014; Ylijoki *et al*., 2011). It is often noted, for example, that whether an evaluation is conducted at a micro- or macro-level significantly affects whether and how quantitative indicators should be used ( Waltman, 2018).

**Figure 2.**
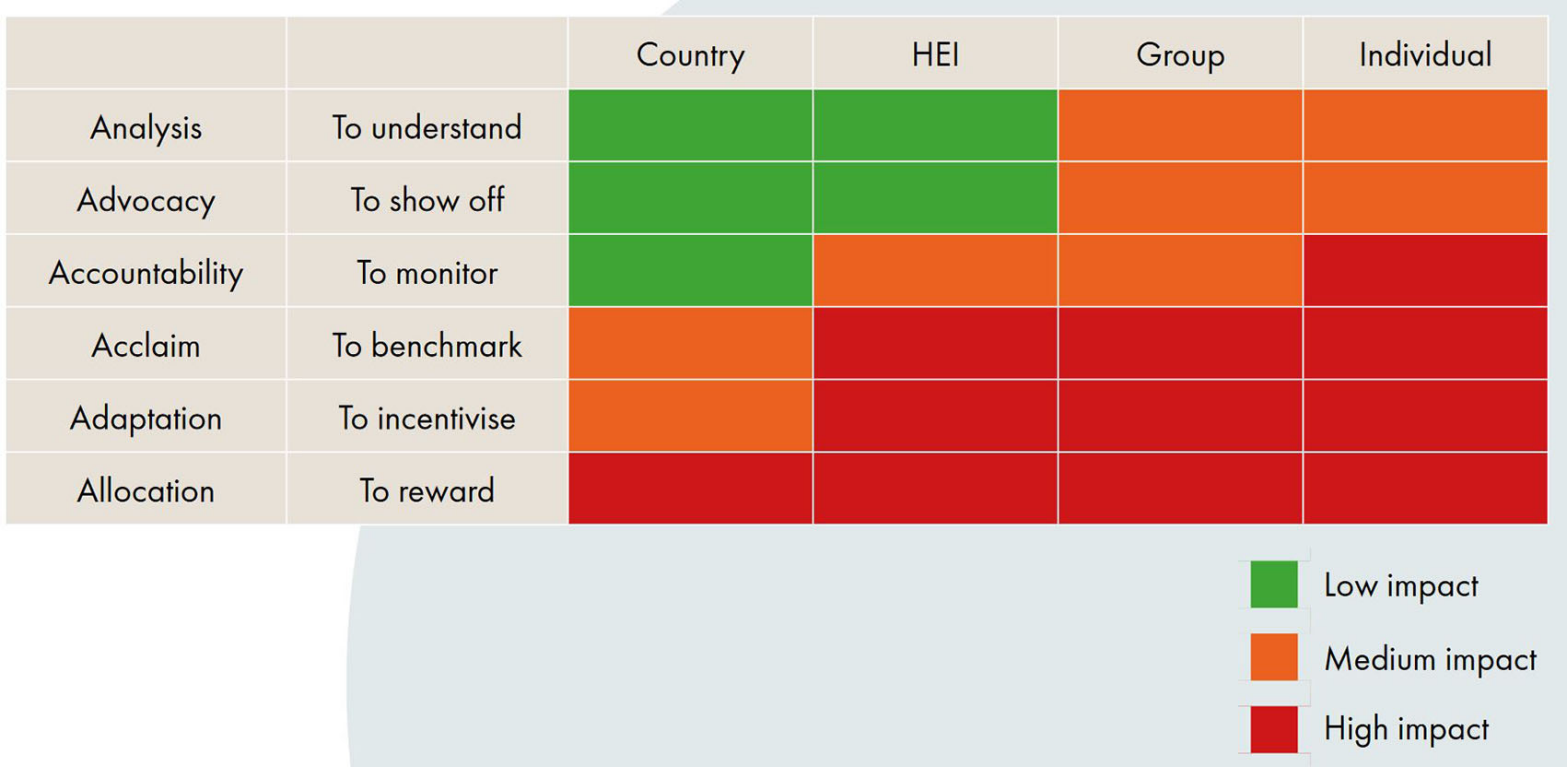
SCOPE ‘Context’ Matrix defining where assessments may have a low/medium/high impact on the assessed entity (from The SCOPE Framework: a five-stage process for evaluating responsibly: https://doi.org/10.26188/21919527.v1).

In terms of purposes, there are six commonly accepted purposes of research assessment: analysis, advocacy, allocation, accountability, acclaim and adaptation ( Parks *et al*., 2019). The meanings of these terms are often contested by stakeholders, so the INORMS REG have provided short interpretive descriptions of each to aid understanding (see Figure 2). Sometimes evaluations can seek to achieve several different purposes, but it is important to specify these in advance and to consider the purpose in conjunction with the entity under evaluation, in order to ensure the evaluation design is appropriate. What works in one context does not necessarily work in another.

To aid this process, the INORMS REG have developed a matrix plotting the six key evaluation purposes against four different entity sizes to highlight how the impact of an assessment varies ( Figure 2). The matrix illustrates that assessments in some settings have more impact on the entity being evaluated and are therefore more ‘high risk’. For example, monitoring a country’s research performance has less impact on the country being evaluated than evaluating an individual researcher for a promotion, and is therefore arguably a lower risk form of assessment. The exact ‘RAG-rating’ of each of these combinations might be debatable, but the matrix provides a useful heuristic to aid evaluators in understanding the dimensionality of research assessment and to ensuring that assessment approaches are context-sensitive.

### 6.3 Options for evaluating

The third stage of SCOPE is to explore all the ***options*** available for evaluating. This stage is a reminder to consider both quantitative and qualitative approaches and consider them in terms of the values and context of the evaluation (see, e.g., Butler, 2007; De Jong *et al*., 2011; Gingras, 2014; Holtrop *et al*., 2020c). The rule of thumb proposed by SCOPE is that quantitative indicators are best reserved for assessing quantitative things: student numbers, money, and citations. In the same way, qualitative approaches are best used for qualitative things: impact and quality. Caution should be taken about using quantitative indicators as a proxy for qualitative things. For example, citation counts are not a suitable proxy for research quality (see, e.g., Aksnes *et al*., 2019).

It is easy to focus on the dangers of quantitative approaches when considering responsible research assessment, and many declarations and principles do so. However, whilst peer-review is considered the gold standard for research evaluation, it is not without its own challenges ( Bornmann & Daniel, 2006; Lee *et al*., 2013; Waltman *et al*., 2023). Recent concerns about increased journal retractions, and the prevalence of so-called ‘predatory’ journals have raised questions about the quality and reproducibility of peer review. Proponents of open research are calling for greater transparency and openness of peer review, and there are equity, diversity and inclusion concerns ( Else & Perkel, 2022).

The truth is that there are limitations to all forms of research assessment, both qualitative and quantitative. For this reason, SCOPE requires evaluators to consider all their assessment options equally. It advocates that in most cases a mixed methods approach is more likely to generate a proportionate and appropriate assessment that will (as with the Hippocratic Oath) first do no harm ( Sugimoto & Larivière, 2018). This will always involve human judgement in some form, and always involve an approximation of the reliability of the assessment, through error bars, list of caveats or limitations, and so on.

Given the many and varied values and contexts which may be evaluated, it is not possible to provide a comprehensive list of options for doing so via the SCOPE framework. However, the guidance promotes the use of alternative evaluation approaches such as those provided by the DORA resource library and the Metrics Toolkit to offer some inspiration.

At this stage it may be helpful to generate several different options given that the Probe stage will ‘stress-test’ these options and may render some unsuitable. The alternative is to consider both the Options and Probe stages together to ensure that no option is developed to such an extent that it becomes difficult to abandon it after being ‘probed’.

### 6.4 Probe deeply

Once options for evaluating have been selected in line with stakeholder values and context and options, the fourth step is to ***probe deeply***. To do this, SCOPE proposes that the evaluator should ask the following four questions of their evaluation:

6.4.1 *Who might the chosen approach discriminate against?*

There is a considerable literature around the biases inherent in all forms of research evaluation as already stated. Demographics most likely fall victim to poor forms of assessment include early-career researchers ( Algra *et al*., 2020), women ( Jappelli *et al*., 2017, 2017; Larivière *et al*., 2013; Sugimoto & Larivière, 2023), intersectional groups ( Bailey, 2018), and those working in non-journal-based disciplines. No evaluation is perfect and, as discussed, there are weaknesses in both qualitative and quantitative forms of assessment. For this reason, it is important to give significant thought to the question as to whether all entities being assessed have equal opportunity to succeed under the evaluation approach selected. If not, what mechanisms might be put in place to mitigate these inherent biases. Much work has been done in this space included the introduction of lotteries to more equitably decide between equally scoring proposals ( Roumbanis, 2019), and the use of Unconscious Bias Observers on promotion panels ( Bonello *et al*., 2017). ‘Evaluating with the evaluated’ (SCOPE’s second principle) and ensuring any consulted stakeholder group is representative, will also go some considerable way to addressing this question.

6.4.2 *How might this approach be gamed?*

A mantra often used by the INORMS REG is that ‘where there is a prize there is a game’. This refers to the fact that where there is a lot at stake in a particular evaluation (reputationally and financially), the entities being evaluated will naturally be incentivised to alter their behaviours in ways that enable them to perform well ( Biagioli & Lippman, 2020). In some cases, this is indeed the purpose of an evaluation: evaluators will seek to assess a particular dimension (e.g., open research) to incentivise it. However, there is a spectrum of responses from evaluated parties to evaluation efforts, from legitimately optimising their activities (e.g., making more outputs open access), to ‘gaming’ their submissions (e.g., only reporting outputs once they’ve been made open access), to outright cheating (fabricating open access data). A strong evaluation should seek to anticipate potential opportunities for gaming with a view to designing them out of the system.

6.4.3 *What might the unintended consequences be?*

In his book, The Tyranny of Metrics, Muller devotes a whole chapter to “the unintended but predictable negative consequences” of poor assessment practices and indicators ( Muller, 2018). He describes some common unintended consequences in terms of goal displacement, short-termism, diminishing utility, rewarding luck, and discouraging risk-taking, innovation and cooperation. Trying to predict the potential harmful consequences of an evaluation approach into which the evaluator has invested much care and effort, is a difficult ask. Whilst it is not always possible to predict unintended consequences, it is important to attempt to do so at both an institutional as well as an individual level (see, e.g., Dahler-Larsen, 2014; Lorenz, 2014; Rijcke *et al*., 2016; Stephan *et al*., 2017; Wellcome Trust, 2020). The use of workshops to design evaluations ‘with the evaluated’, where the evaluated act as ‘critical friends’ is one useful way of identifying some of the unintended consequences before time and expense is invested into running the evaluation. However, it should be accepted that some consequences are not always predictable, and this is a question that should be returned to at the ‘Evaluate’ stage of SCOPE.

6.4.4 *Does the cost of measuring outweigh the benefit?*

Another of Mueller’s unintended consequences of evaluation is the significant costs that may be incurred in both running and interpreting the assessment ( Muller, 2018). SCOPE is clear that the cost, including the workload, stress, and finances, should be proportional to the aims and anticipated outcomes of the evaluation ( Sawczak, 2018). Given the strongest evaluations usually consist of a mixed methods approach involving some element of human judgement, the cost of an evaluation can quickly escalate.

A current case in point is the reported cost of running the 2021 UK Research Excellence Framework which came in at £471 million ( Research England *et al*., 2023). This is only 3–4% of the block-grant funding linked to its outcomes; however, it is almost double the cost of the 2014 exercise ( Else, 2015), which was in turn three times higher than the 2008 exercise ( Sayer, 2015). The Joint UK HE Funding bodies have explicitly stated an intention to reduce the cost of the exercise in 2028. Similarly, the Danish government recently announced that they would discontinue the updating of the national bibliometric research indicator due to the cost and burden not being commensurate with the benefit ( Uddannelses- og Forksningsministeriet, 2021).

There is no ‘correct answer’ when it comes to the cost:benefit ratio of an evaluation. However, as with all business decisions, those making the investment need to be reassured of an appropriate return. While evaluations can be extremely beneficial to generate intelligence, evidence, improve efficiencies or identify gaps, these benefits are only realized if the evaluation is designed in a way that provides usable outcomes at a reasonable cost.

### 6.5 Evaluate your evaluation

The fifth, and final stage of SCOPE is to ***evaluate your evaluation***. After conducting an evaluation, it is important to check if it reached its aims, if the results are useful, and if the evaluation approach brought new insights to what was being evaluated. Did the evaluation cause unintended consequences not foreseen at the Probe stage? If so, they should be considered when interpreting the results and addressed prior to future assessments. Even where an approach proved to be successful, it should be kept in mind that the tools available to undertake an evaluation (e.g., the data sources and indicators available) as well as values, missions, and strategies, are subject to change.

This last step of SCOPE is often overlooked but is of vital importance. Stufflebeam even considers meta-evaluation – the evaluation of evaluations – as a professional obligation of evaluators ( Stufflebeam, 2001; see also, Scriven, 2009). In addition to any immediate post-assessment evaluation, established evaluations should also be re-evaluated at regular intervals to ensure they are still in alignment with what is valued about the entity under evaluation, and does not result in any unintended consequences that may require an adjustment to the evaluation. One of the strengths of SCOPE is that it can be used to both design new evaluations, and to assess existing evaluations. Thus the ‘E’ of SCOPE is really an invitation to run through the SCOPE process again to assess the evaluation that has been designed and implemented.

Whilst SCOPE is presented as a linear, step-by-step, process, it is rather more iterative in practice. Each stage of SCOPE might send the evaluator back to a previous stage to reconsider a prior decision. For example, the unintended consequences unearthed at the Probe stage might cause the evaluator to consider whether the Options they have chosen are sensible, or even whether the evaluation is suitable for a particular Context. Ultimately, as long as each stage of SCOPE is considered in the design of an evaluation, it has a strong chance of being an appropriate and proportionate assessment.

## 7 Use cases

The SCOPE framework is in wide usage by a range of organisations globally. Recent examples include the use of SCOPE by Indian funding agencies to develop assessment mechanisms ( Suchiradipta *et al*., 2023), by Colombian research professionals to develop a responsible assessment policy ( Pallares *et al*., 2023), by Finnish research managers to create an institutional policy on assessment of researchers ( University of Turku, n.d.) and by UK research leaders to develop an approach to assessing research culture ( Davies & Fadhel, 2023). Example case studies are regularly added to the SCOPE webpage. This section provides four case studies to demonstrate how it has been used by different types of organisations to develop evaluations at different levels of granularity and in different settings.

### 7.1 Case Study: Emerald Publishing, UK

7.1.1 *Background*

Emerald Publishing is a global scholarly publisher committed to equity, diversity, and inclusion. As such they were keen to start monitoring and incentivising greater diversity and representation on the editorial boards of their scholarly journals. Being aware of the sensitivities around this, they used the SCOPE framework to explore how they might do so ( Gadd & Himanen, 2021a).

7.1.2 *Process*

The publisher firstly ran a 90-minute ***Start with what you value*** workshop with ten editors and editorial board members and supported by the INORMS REG. This explored ‘what might a diverse editorial board look like’ and ‘what aspects of diversity actually benefit a journal’ in the agreed ***context*** of ‘incentivising’ greater diversity in editorial boards. Given the SCOPE principle of evaluating only where necessary, a discussion was held as to whether the valued dimensions of diversity should be enabled rather than evaluated. ***Options*** for evaluating this value in these contexts were discussed with Emerald first presenting some examples of what can be assessed using existing systems and approaches.

A second 90-minute workshop was then run to explore ***Options*** in greater detail with the Emerald Publishing Editorial and Rights/Legal team members. The options were ***probed*** using the four key ***probe*** questions at the same time.

7.1.3 *Outcomes*

A key learning point from the ***values*** stage was that the editorial board members’ views of diversity included subject diversity, diversity in the way knowledge is/can be disseminated as well as regional diversity. Editorial Board members also felt that a commitment to diversity should be taken as part of a wider review of editorial board processes and policies, e.g., dormancy in editorial boards and whether boards would benefit from shorter-term appointments. Also, they felt that diversity within editorial boards was necessarily affected by the broader challenges around the prevailing model of creating scientific knowledge (e.g., the use of unpaid editorial positions) and global inequality more broadly.

Considering the ***context*** of ‘incentivisation’ led to interesting discussions as to whether this value needed enabling or evaluating. Interestingly, the workshop members felt it needed both, and that some sort of ‘badges’ or external signifier that the journal was at least committed to improving diversity, would be welcomed. Another important question asked who was being incentivised in this context: Emerald Publishing, editors and/or editorial board members? Ultimately it was agreed that editors should be the focus of any enabling and evaluating activity, but the relationship between publisher and editors is a carefully balanced one.

While a mixed evaluate-and-enable approach was agreed upon, many of the actions and ideas that came from the workshops were focussed on enabling diversity. ***Options*** floated included both paying editorial board members to allow less-well-funded scholars to participate and introducing a Diversity Editorial Pledge whereby editors could be rewarded for a commitment to diversity. ***Probing*** these ***options*** generated questions around the sensitivities and challenges associated with monitoring diversity-related data.

The SCOPE process resulted in a range of short, medium, and long-term actions for Emerald Publishing to pursue. The immediately actionable items included building expectations around diversity into contracts/job descriptions for Editors; making diversity a rolling agenda point for Editorial meetings and reporting on progress around diversity in Editorial Advisory Board (EAB) meetings; and encouraging editors and EABs to identify their own success EDI indicators in line with their values.

### 7.2 Case Study: The UK HE Joint Funding Bodies ‘Future Research Assessment Programme’

7.2.1 *Background*

Research England, the Scottish Funding Council, the Northern Ireland Department for the Economy, and the Higher Education Funding Council for Wales commissioned the Future Research Assessment Programme (FRAP) with a view to designing a new national research assessment exercise in the UK. The outcomes of the current system are used to inform the allocation of quality-related research funding to UK universities and provide accountability for public investment in research. It was felt that without a proper framework such a review could focus narrowly on making minor adjustments to the existing system (‘evolution’) or solely rely on learning from other international research assessment approaches (‘reproduction’) when what the funding bodies really sought was a root-and branch review (possible ‘revolution’). It was felt that the SCOPE framework offered a structure for thinking about such a review, providing “the perfect brief to be radical” ( Gadd & Himanen, 2021b, p. 2).

7.2.2 *Process*

**Figure 3.**
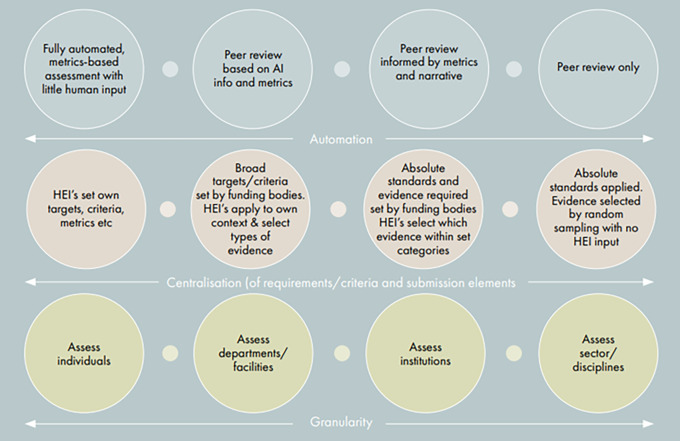
Spectra used to identify stakeholder appetites for different evaluation variables (from INORMS SCOPE Case Study: The UK Higher Education Funding Bodies - Developing a new national research evaluation system: https://inorms.net/wp-content/uploads/2021/11/inorms-scope-case-study-uk-he-funding-bodies-final.pdf).

The use of SCOPE by the funding bodies was unmediated by the INORMS REG ( Gadd & Himanen, 2021b). The FRAP team were keen to ***start with what was valued*** about the research system. Deploying the ‘Evaluate with the evaluated’ principle, they held a series of round-table events with different stakeholder groups. To create a healthy ecosystem, it was important to the team to not only start with what was valued, but to also agree which of those valued things should be evaluated. Understanding the ***contexts*** in which the national research evaluation exercise should take place was an important step in the process. The REF exercise that ran in 2021 served a wide range of purposes and the team sought to identify which were non-negotiable. The FRAP team were inspired by the ***options*** stage of exploring both qualitative and quantitative options to develop a set of spectra to understand the community's appetite for different variables, for example, around the degree of automation, centralisation, granularity, and frequency (see Figure 3).

Of particular interest to the funding bodies was to ***probe*** for unintended consequences and perverse incentives. The cost-benefit of the exercise was explored in a dedicated assessment ( Neto *et al*., 2023).

7.2.3 *Outcomes*

The REF 2028 Initial Decisions ( Research England *et al*., 2023) show many of the hallmarks of a SCOPE-led assessment. The principle of ‘evaluating with the evaluated’ and starting with what was ***valued*** is clearly in evidence and the principle of’drawing on evaluation expertise’ was fulfilled by utilising specialists to produce reports on metrics ( Curry *et al*., 2022), artificial intelligence ( Thelwall *et al*., 2022) and a ‘real-time REF review’ to support the process ( Manville *et al*., 2021). The clear articulation of the purposes of the next REF specifies the ***context*** and the ***options*** carefully balance both qualitative and quantitative measures in an effort to reduce burden. The Initial Decisions document makes frequent reference to the need to mitigate unintended consequences particularly to under-represented groups. Further consultation is afoot, again on the principle of ‘Evaluating with the Evaluated’ to ensure no foreseeable but unintended consequences are at play.

### 7.3 Case Study: Newcastle University, UK ^1^

7.3.1 *Background*

To support Newcastle University’s work on enhancing their research culture, they used the SCOPE framework to develop a set of research culture Key Performance Indicators (KPIs) for the University’s Research Strategy and a basket of measures for their ‘Research Culture Index’.

7.3.2 *Process*

Using the SCOPE framework and supported by the INORMS REG, the University held an initial community workshop with around 80 colleagues (both academic and professional services colleagues) and postgraduate students to identify what people ***valued*** in a positive research culture. They sought to understand both what a positive research culture ‘looked and felt like’ to fully understand how it might enhance the research community’s experiences in relation to doing great research. The resulting values were then tested and refined at a subsequent smaller workshop of some of the attendees.

Once agreed, a smaller facilitated focus group was held to generate a series of ***options*** of measures for evaluation within the ***context*** of’monitoring’ as KPIs. The options were then ***probed*** for any unintended consequences. The resulting set of measures were shared with colleagues with relevant expertise within the University to check for feasibility and viability in the ***context*** of different Faculty-based disciplines and given the practicalities of the University’s research reporting systems.

7.3.3 *Outcomes*

The first high-level workshop identified four key attributes of a positive research culture in terms of:

• Collaboration and collegiality
• The freedom to explore and grow
• Fairness and inclusion
• Openness and integrity

The second workshop explored the dimensions (or ‘sub-values’) of these attributes that constituted the’look and feel’ of a positive research culture. These included strong support for the careers of others, a sense of belonging, and increasing empowerment and satisfaction. These sub-values more easily lent themselves to the development of specific ***options*** for monitoring improvement.

Seventeen options were ultimately selected after a number were excluded during the ***probe*** stage. One example of an excluded measure was ‘Bullying and harassment reporting.’ This was excluded as it would only reflect reporting rather than occurrence, and it was unclear whether the measure should increase or decrease. For example, an increase in reporting could be interpreted as a positive if people feel safer and more supported to report, but also a negative if it captures increasing incidence. The final outcome of the process was a ‘Research Culture Index’ with seventeen dimensions and some of these informed the revision of the University’s Research Strategy KPIs.

### 7.4 Case Study: University of Alberta, Canada ^2^

7.4.1 *Background*

The University of Alberta, in Edmonton, Canada, has been exploring ways to implement responsible research evaluation approaches throughout the University. Particularly their Research Impact Librarians were interested in learning how to conduct research assessment more effectively and more equitably. Upon learning of the SCOPE framework, they identified the need to provide training on this approach to various members of their community.

7.4.2 *Process*

It was determined to hold two consecutive workshops assisted by the INORMS REG that focused on the exploration and then implementation of the SCOPE framework, with the goal to increase capacity within the University of Alberta to develop, evaluate and refine responsible research assessments.

The first workshop was more didactic in nature as many attendees were not yet familiar with the SCOPE framework. As such, this 90-minute session focused on the principles and stages within the SCOPE framework, providing the knowledge and understanding behind each stage of the process. This workshop was attended by a broad range of individuals including senior university leadership, representatives from various faculties and departments, and library information specialists.

The second 2-hour workshop focused on the direct implementation and application of the SCOPE framework with a smaller subset of individuals who routinely develop or participate in research assessment. Since the University was working to develop better assessment of knowledge mobilisation (KM), this was used as the working example. For each stage of the SCOPE framework various small group exercises or group discussions were held to explore the aspects of KM that were ***valued*** the most (impact in policy, uptake by community, scientific knowledge, etc); in which ***context*** they sought to assess KM (advocacy of the organization, accountability of departments, etc.); what were the ***options*** they had, or could develop, to assess KM (collaborations, policy changes, publications, commercialisation, etc.); how they could ***probe*** these options to determine if there was bias or discrimination that needs to be considered or addressed, and finally how they could thoughtfully ***evaluate*** their assessment to determine if it met the principles of the SCOPE framework.

7.4.3 *Outcome*

The University of Alberta found these workshops increased both knowledge and capacity in the responsible research assessment. While KM was used a case example during the workshop, it was acknowledged that development of robust framework would need to be co-developed with those being evaluations and should also involve reaching out to others who were heavily invested in the assessment of KM, such as Research Impact Canada, to draw on available expertise. The University of Alberta now has plans to use SCOPE in ongoing development of multiple assessments and in the re-evaluation of previously established processes.

## 8 Discussion and conclusions

The SCOPE framework seeks to support evaluators in any and every research setting to implement the many complementary principles of RRA in the design and delivery of their evaluation approaches. In this way it bridges the gap between principles and practice. However, SCOPE does not simply translate existing principles into practice, but provides additional considerations not always addressed by principles of RRA, such as evaluating with the evaluated, evaluating only where necessary, starting with what is valued and probing for unintended consequences. Thus SCOPE, with its focus on implementation, plays a specific role in the delivery of RRA.

The use cases presented show the value of SCOPE across a range of settings. This highlights one of the strengths of SCOPE, namely, that it is widely applicable and enables both quantitative and qualitative assessments for any purpose, at any level, and any discipline and can be used by any evaluator with any background (assuming of course, that they draw on appropriate expertise as required by principle three). To develop a framework with such wide applicability has necessitated it to take a very high-level, somewhat simplified, approach to research assessment. Indeed, the whole framework can be presented in a one-page overview (see Figure 1). This is both a strength and a weakness.

Much of the feedback received is that the beauty of SCOPE lies in its simplicity. The main principles and stages can be communicated and understood in a few minutes. It can also be used with or without assistance from the INORMS REG as evident from the use cases. This is probably why it has captured the imagination of the global research community and been so widely adopted. However, as can be seen by the full SCOPE guide there is a lot more underneath the simple heuristics to be explored and understood ( International Network Of Research Management Societies-Research Evaluation Group, 2021). An evaluator that has sought to apply SCOPE without drawing on appropriate evaluation expertise may misinterpret some of the steps and claim they have a ‘SCOPE-compliant’ evaluation where this might not be the case.

Other feedback sometimes received is that the framework is common sense and aligned with existing practice. Whilst the team would agree with the former, continued evidence of poorly designed research assessments gives the lie to the latter. SCOPE is simple but is not universally applied. Were each of the stages of SCOPE properly applied under its three principles, the existence of problematic research evaluations would be greatly diminished. What might look like common sense at first is, in reality, a series of deep and fundamental questions enabling both the evaluators and the evaluated to reflect on their practices and their assumptions. This reflection leads to an explicit definition of the values that are the foundation for an evaluation and focuses attention on potential biases and weaknesses in the evaluation design, which may not ordinarily be given their due attention. Thus, where evaluation may previously have been an implicit part of publishing, hiring, budgeting, etc., SCOPE puts a focus on evaluation in its own right.

One of the strengths of SCOPE is that in addition to enabling the design of responsible research assessments, it can act as a training framework for research evaluators. Many more professionals (research managers, planners, funders, librarians, and publishers) are being called upon to design or evaluate research with no formal training, nor the capacity or opportunity to undertake any. Many researchers who have had to participate in some form of evaluation as part of their roles (journal peer review, recruitment, etc.,) might feel that this knowledge is transferrable to other forms of evaluation without recognising some of the differences between various forms of assessment. SCOPE is simple enough and accessible enough to provide a framework for a deeper understanding of responsible research assessment practice and could support the greater professionalisation of research evaluation.

As demonstrated in this paper each of the three principles and the five stages in the SCOPE framework are rooted firmly in the existing research literature, whilst consolidating and expanding on this evidence with lessons learned from experience. Whilst RRA began as a series of objections against data-driven research evaluations, the SCOPE framework provides evaluators with a more positive, comprehensive, and practical approach to all forms of research assessment. It is offered up to the community as a useful tool in the toolbox of all research evaluators.

## Author Contributions

• Conceptualization: all
• Investigation: all
• Methodology: all
• Project administration: Himanen, Gauffriau, Gadd
• Supervision: Gadd
• Writing – original draft: Himanen, Conte, Gauffriau, Gadd
• Writing – review & editing: all

## Data Availability

All data underlying the results are available as part of the article and no additional source data are required.

## Footnotes

^1^
The case description is based on INORMS REG members’ meetings with Sarah Whalley, Research Strategy Manager at Newcastle University.

^2^
The case description is based on INORMS REG members’ meetings with Thane Chambers, Librarian at University of Alberta.

## References

[ref1] Aagaard K : How incentives trickle down: Local use of a national bibliometric indicator system. *Sci. Public Policy.* 2015;42(5):725–737. 10.1093/scipol/scu087

[ref2] Agate N Kennison R Konkiel S : The transformative power of values-enacted scholarship. *Humanities and Social Sciences Communications.* 2020;7(1):165. 10.1057/s41599-020-00647-z

[ref3] Aksnes DW Langfeldt L Wouters P : Citations, Citation Indicators, and Research Quality: An Overview of Basic Concepts and Theories. *SAGE Open.* 2019;9(1):215824401982957. 10.1177/2158244019829575

[ref4] Algra A Koopman I Snoek R : How young researchers can re-shape the evaluation of their work. Nature Index News. 2020. Reference Source

[ref5] Aubert Bonn N De Vries RG Pinxten W : The failure of success: Four lessons learned in five years of research on research integrity and research assessments. *BMC. Res. Notes.* 2022;15(1):309. 10.1186/s13104-022-06191-0 36153631 PMC9509645

[ref6] Aubert Bonn N Pinxten W : Advancing science or advancing careers? Researchers’ opinions on success indicators. *PLoS One.* 2021a;16(2):e0243664. 10.1371/journal.pone.0243664 33571992 PMC7878066

[ref7] Aubert Bonn N Pinxten W : Rethinking success, integrity, and culture in research (part 1)—A multi-actor qualitative study on success in science. *Research Integrity and Peer Review.* 2021b;6(1):1. 10.1186/s41073-020-00104-0 33441187 PMC7807516

[ref8] Bailey M : On misogynoir: Citation, erasure, and plagiarism. *Fem. Media Stud.* 2018;18(4):762–768. 10.1080/14680777.2018.1447395

[ref9] Ballantyne N : Epistemic Trespassing. *Mind.* 2019;128(510):367–395. 10.1093/mind/fzx042

[ref10] Benedictus R Miedema F Ferguson MWJ : Fewer numbers, better science. *Nature.* 2016;538(7626):453–455. 10.1038/538453a 27786219

[ref11] Biagioli M Lippman A : *Gaming the metrics: Misconduct and manipulation in academic research.* The MIT Press;2020. 10.7551/mitpress/11087.001.0001

[ref12] Birukou A Wakeling JR Bartolini C : Alternatives to Peer Review: Novel Approaches for Research Evaluation. *Front. Comput. Neurosci.* 2011;5. 10.3389/fncom.2011.00056 22174702 PMC3237011

[ref13] Blomkamp E : The Promise of Co-Design for Public Policy. *Aust. J. Public Adm.* 2018;77(4):729–743. 10.1111/1467-8500.12310

[ref14] Bonello E Coombs H Dessent C : *Unconscious bias observer scheme: DEVELOPMENT OF A NOVEL UNCONSCIOUS BIAS OBSERVER SCHEME AT CHEMISTRY@YORK.* Sheffield: LGBTSTEMinar;2017. Reference Source

[ref15] Bornmann L Daniel H-D : Potential sources of bias in research fellowship assessments: Effects of university prestige and field of study. *Research Evaluation.* 2006;15(3):209–219. 10.3152/147154406781775850

[ref16] Butler L : What Happens When Funding is Linked to Publication Counts? Moed HF Glänzel W Schmoch U , editors. *Handbook of Quantitative Science and Technology Research.* Kluwer Academic Publishers;2005; (pp.389–405). 10.1007/1-4020-2755-9_18

[ref17] Butler L : Assessing university research: A plea for a balanced approach. *Sci. Public Policy.* 2007;34(8):565–574. 10.3152/030234207X254404

[ref18] Campbell DT : Assessing the impact of planned social change. *Eval. Program Plann.* 1979;2(1):67–90. 10.1016/0149-7189(79)90048-X

[ref19] Curry S Gadd E Wilsdon J : *Harnessing the Metric Tide: Indicators, infrastructures &amp; priorities for UK responsible research assessment.* Research on Research Institute;2022;11014215 Bytes. 10.6084/M9.FIGSHARE.21701624.V2

[ref20] Dahler-Larsen P : Constitutive Effects of Performance Indicators: Getting beyond unintended consequences. *Public Manag. Rev.* 2014;16(7):969–986. 10.1080/14719037.2013.770058

[ref21] Davies C Fadhel S : *Using SCOPE to select metrics to track improvements in Research Culture: Interim reflections.* University of Leeds;2023. Reference Source

[ref22] De Jong SPL Van Arensbergen P Daemen F : Evaluation of research in context: An approach and two cases. *Research Evaluation.* 2011;20(1):61–72. 10.3152/095820211X12941371876346

[ref23] Else H : REF 2014 cost almost £250 million. *Times Higher Education.* 2015. Reference Source

[ref24] Else H Perkel JM : The giant plan to track diversity in research journals. *Nature.* 2022;602(7898):566–570. 10.1038/d41586-022-00426-7 35197624

[ref25] European University Association, Science Europe, European Commission Stroobants K : Agreement on reforming research assessment. 2022; (p.23). Reference Source

[ref26] Feldman Z Sandoval M : Metric Power and the Academic Self: Neoliberalism, Knowledge and Resistance in the British University. *TripleC: Communication, Capitalism & Critique. Open Access Journal for a Global Sustainable Information Society.* 2018;16(1):214–233. 10.31269/triplec.v16i1.899

[ref27] Fochler M De Rijcke S : Implicated in the Indicator Game? An Experimental Debate. *Engag. Sci. Technol. Soc.* 2017;3:21–40. 10.17351/ests2017.108

[ref28] Gadd E : University rankings need a rethink. *Nature.* 2020;587(7835):523–523. 10.1038/d41586-020-03312-2 33235367

[ref29] Gadd E : Mis-measuring our universities: Why global university rankings don’t add up [Preprint]. *SocArXiv.* 2021. 10.31235/osf.io/gxbn5 PMC8459771 34568739

[ref30] Gadd E Himanen L : INORMS SCOPE Case Study: Emerald Publishing: Evaluating the diversity of editorial boards. 2021a; (p.5). Reference Source

[ref31] Gadd E Himanen L : INORMS SCOPE Case Study: The UK Higher Education Funding Bodies: Developing a new national research evaluation system. 2021b; (p.5). Reference Source

[ref32] Geuna A Martin BR : University Research Evaluation and Funding: An International Comparison. *Minerva.* 2003;41(4):277–304. 10.1023/B:MINE.0000005155.70870.bd

[ref33] Gewin V : Has the ‘great resignation’ hit academia?. *Nature.* 2022;606(7912):211–213. 10.1038/d41586-022-01512-6 35641675

[ref34] Gingras Y : Criteria for evaluating indicators. *Beyond bibliometrics: Harnessing multidimensional indicators of scholarly impact.* The MIT Press;2014; (pp.109–125).

[ref35] Gläser J Laudel G : Evaluation Without Evaluators. Whitley R Gläser J , editors. *The Changing Governance of the Sciences.* Netherlands: Springer;2007; Vol.26: pp.127–151. 10.1007/978-1-4020-6746-4_6

[ref36] Hallonsten O : Stop evaluating science: A historical-sociological argument. *Soc. Sci. Inf.* 2021;60(1):7–26. 10.1177/0539018421992204

[ref37] Hicks D Wouters P Waltman L : Bibliometrics: The Leiden Manifesto for research metrics. *Nature.* 2015;520(7548):429–431. 10.1038/520429a 25903611

[ref38] Holtrop T Hessels L Prins A : Evaluative Inquiry I: Academic value is more than performance. *Leiden Madtrics.* 2020a. Reference Source

[ref39] Holtrop T Hessels L Prins A : Evaluative Inquiry II: Evaluating research in context. *Leiden Madtrics.* 2020b. Reference Source

[ref40] Holtrop T Hessels L Prins A : Evaluative Inquiry III: Mixing methods for evaluating research. *Leiden Madtrics.* 2020c. Reference Source

[ref41] International Network Of Research Management Societies-Research Evaluation Group: *The SCOPE Framework: A five-stage process for evaluating research responsibly.* International Network of Research Management Societies;2021;668558 Bytes. 10.26188/21919527.V1

[ref42] Jappelli T Nappi CA Torrini R : Gender effects in research evaluation. *Res. Policy.* 2017;46(5):911–924. 10.1016/j.respol.2017.03.002

[ref43] Konkiel S : Approaches to creating ‘humane’ research evaluation metrics for the humanities. *Insights the UKSG Journal.* 2018;31:44. 10.1629/uksg.445

[ref44] KPMG: Health workforce equity, diversity and inclusion: Taking deliberate actions to develop inclusive and equitable workplace cultures. 2022. Reference Source

[ref45] Lamont M Huutoniemi K : Comparing Customary Rules of Fairness: Evaluative Practices in Various Types of Peer Review Panels. Camic C Gross N Lamont M , editors. *Social knowledge in the making.* University of Chicago Press;2011.

[ref46] Lane J Largent M Rosen R : Science Metrics and Science Policy. Cronin B Sugimoto CR , editors. *Beyond bibliometrics: Harnessing multidimensional indicators of scholarly impact.* The MIT Press;2014; (pp.397–411).

[ref47] Larivière V Ni C Gingras Y : Bibliometrics: Global gender disparities in science. *Nature.* 2013;504(7479):211–213. 10.1038/504211a 24350369

[ref48] Lebel J McLean R : A better measure of research from the global south. *Nature.* 2018;559(7712):23–26. 10.1038/d41586-018-05581-4 29973734

[ref49] Lee CJ Sugimoto CR Zhang G : Bias in peer review. *J. Am. Soc. Inf. Sci. Technol.* 2013;64(1):2–17. 10.1002/asi.22784

[ref50] Leydesdorff L Wouters P Bornmann L : Professional and citizen bibliometrics: Complementarities and ambivalences in the development and use of indicators—a state-of-the-art report. *Scientometrics.* 2016;109(3):2129–2150. 10.1007/s11192-016-2150-8 27942086 PMC5124044

[ref51] Lorenz C : Fixing the facts: The rise of new public management, the metrification of “quality” and the fall of the academic professions. *Moving the Social.* 2014;52:5–26.

[ref52] Manville C d’Angelo C Culora A : *Understanding perceptions of the Research Excellence Framework among UK researchers: The Real-Time REF Review.* RAND Corporation;2021;140. Reference Source

[ref53] Merton RK : The normative structure of science. Merton RK Storer NW , editors. *The Sociology of Science: Theoretical and Empirical Investigations.* The University of Chicago Press;1973.

[ref54] Moher D Bouter L Kleinert S : The Hong Kong Principles for assessing researchers: Fostering research integrity. *PLoS Biol.* 2020;18(7):e3000737. 10.1371/journal.pbio.3000737 32673304 PMC7365391

[ref55] Moher D Naudet F Cristea IA : Assessing scientists for hiring, promotion, and tenure. *PLoS Biol.* 2018;16(3):e2004089. 10.1371/journal.pbio.2004089 29596415 PMC5892914

[ref56] Morrish L : Why the audit culture made me quit. *Times Higher Education.* 2017. Reference Source

[ref57] Moser SC : Can science on transformation transform science? Lessons from co-design. *Curr. Opin. Environ. Sustain.* 2016;20:106–115. 10.1016/j.cosust.2016.10.007

[ref58] Muller J : *The Tyranny of Metrics.* Princeton University Press;2018. 10.23943/9781400889433

[ref59] Neto A Vingre A D’Hont J : REF 2021 Cost Evaluation: Final report. 2023; (p.78). Reference Source

[ref60] Pallares C Chalela S Tejada MA : The Colombian responsible metrics Project: Towards a Colombian institutional, methodological instrument for research assessment. *DORA Blog.* 2023. Reference Source

[ref61] Parks S Rodriguez-Rincon D Parkinson S : *The changing research landscape and reflections on national research assessment in the future (RR-3200-UKRI).* RAND Corporation;2019;204. Reference Source

[ref62] Parr C : Imperial College professor Stefan Grimm ‘was given grant income target.’ *Times Higher Education.* 2014. Reference Source

[ref63] Puuska H-M : *Scholarly Publishing Patterns in Finland: A comparison of disciplinary groups.* Tampere University;2014. Reference Source

[ref64] Research England, Scottish Funding Council, Higher EducationFundng Council for Wales, & Department for the Economy, Northern Ireland: Research Excellence Framework 2028: Initial decisions and issues for further consultation (REF 2028/23/01; p. 34). 2023. Reference Source

[ref65] Rijcke SD Wouters PF Rushforth AD : Evaluation practices and effects of indicator use—A literature review. *Research Evaluation.* 2016;25(2):161–169. 10.1093/reseval/rvv038

[ref66] Roumbanis L : Peer Review or Lottery? A Critical Analysis of Two Different Forms of Decision-making Mechanisms for Allocation of Research Grants. *Sci. Technol. Hum. Values.* 2019;44(6):994–1019. 10.1177/0162243918822744

[ref67] Saenen B Hatch A Curry S : *Reimagining Academic Career Assessment: Stories of innovation and change.* DORA; European University Association; SPARC Europe;2021;47. Reference Source

[ref68] Saenen B Morais R Gaillard V : *Research Assessment in the Transition to Open Science: 2019 EUA Open Science and Access—Survey Results.* European University Association;2019;48. Reference Source

[ref69] San Francisco Declaration on Research Assessment:2012. Reference Source

[ref70] Sawczak K : The hidden costs of research assessment exercises: The curious case of Australia. *LSE Impact Blog.* 2018. Reference Source

[ref71] Sayer D : Why did REF2014 cost three times as much as the RAE? Hint: It’s not just because of the added impact element. *LSE Impact Blog.* 2015. Reference Source

[ref72] Scriven M : Meta-Evaluation Revisited. *Journal of MultiDisciplinary Evaluatio.* 2009;6(11):iii–viii.

[ref73] Steen M Manschot M De Koning N : Benefits of Co-design in Service Design Projects. *Int. J. Des.* 2011;5(2):53–60.

[ref74] Stephan P Veugelers R Wang J : Reviewers are blinkered by bibliometrics. *Nature.* 2017;544(7651):411–412. 10.1038/544411a 28447652

[ref75] Stufflebeam DL : The Metaevaluation Imperative. *Am. J. Eval.* 2001;22(2):183–209. 10.1177/109821400102200204

[ref76] Suchiradipta B Koley M Bharadwa J : Workshop on Research Assessment Practices in Indian Funding Agencies. *Journal of Science Policy & Governance.* 2023;22(1). 10.38126/JSPG220110

[ref77] Sugimoto CR Larivière V : *Measuring research: What everyone needs to know.* Oxford University Press;2018.

[ref78] Sugimoto CR Larivière V : *Equity for women in science: Dismantling systemic barriers to advancement.* Harvard University Press;2023.

[ref79] Thelwall M Kousha K Wouters P : The metric tide: Literature review. 2015. 10.13140/RG.2.1.5066.3520

[ref80] Thelwall M Kousha K Abdoli M : Can REF output quality scores be assigned by AI? Experimental evidence. 2022. 10.48550/ARXIV.2212.08041

[ref81] Uddannelses- og Forksningsministeriet: Aftale mellem regeringen Socialdemokratiet), Dansk Folkeparti, Socialistisk Folkeparti, Radikale Venstre, Enhedslisten, Det Konservative Folkeparti, Nye Borgerlige, Frie Grønne, Liberal Alliance, Alternativet og Kristendemokraterne om: Basismidler til forskning. 2021. Reference Source

[ref82] University of Turku: *Policy for Responsible Assessment of Research and Researcher.* University of Turku;n.d.;8. Reference Source

[ref83] Van Leeuwen T : Descriptive Versus Evaluative Bibliometrics: Monitoring and Assessing of National R&D Systems. Moed HF Glänzel W Schmoch U , editors. *Handbook of Quantitative Science and Technology Research.* Netherlands: Springer;2004; (pp.373–388). 10.1007/1-4020-2755-9_17

[ref84] Van Raan AFJ : Fatal attraction: Conceptual and methodological problems in the ranking of universities by bibliometric methods. *Scientometrics.* 2005;62(1):133–143. 10.1007/s11192-005-0008-6

[ref85] Waltman L : Responsible metrics: One size doesn’t fit all. *CWTS Blog.* 2018. Reference Source

[ref86] Waltman L Kaltenbrunner W Pinfield S : How to improve scientific peer review: Four schools of thought. *Learned Publishing.* 2023;36(3):334–347. 10.1002/leap.1544 38504796 PMC10946616

[ref87] Watermeyer R Derrick GE Borras Batalla M : Affective auditing: The emotional weight of the research excellence framework. *Research Evaluation.* 2023;31(4):498–506. 10.1093/reseval/rvac041

[ref88] Wellcome Trust: *What Researchers Think About the Culture They Work In.* Wellcome Trust;2020;51. Reference Source

[ref89] Wilsdon J Allen L Belfiore E : The metric tide: Report of the independent review of the role of metrics in research assessment and management. 2015. 10.13140/rg.2.1.4929.1363

[ref90] Ylijoki O-H Lyytinen A Marttila L : Different research markets: A disciplinary perspective. *High. Educ.* 2011;62(6):721–740. 10.1007/s10734-011-9414-2

