# Supplementary material for: The SCOPE framework – implementing ideals of responsible research assessment
Source: F1000Res. 2024 May 17;12:1241. Originally published 2023 Sep 28. [Version 2] doi: 10.12688/f1000research.140810.2 (PMC11134161; doi:10.12688/f1000research.140810.2)
Supplement: Peer review history

# Reviewer report: Reviewer response for version 2

**DOI:** 10.5256/f1000research.165965.r279711

**Published:** 2024-05-28

**Authors:** Sven E. Hug

**Affiliations:** 1 Department of Psychology, University of Zurich, Zürich, Switzerland

I'd like to thank the authors for taking the time to consider and address my comments. I have no further comments.

Is the rationale for developing the new method (or application) clearly explained?

Yes

Is the description of the method technically sound?

Yes

Are the conclusions about the method and its performance adequately supported by the findings presented in the article?

Partly

If any results are presented, are all the source data underlying the results available to ensure full reproducibility?

No source data required

Are sufficient details provided to allow replication of the method development and its use by others?

Yes

Reviewer Expertise:

Research evaluation, bibliometrics, peer review

I confirm that I have read this submission and believe that I have an appropriate level of expertise to confirm that it is of an acceptable scientific standard.

# Reviewer report: Reviewer response for version 2

**DOI:** 10.5256/f1000research.165965.r279712

**Published:** 2024-05-27

**Authors:** Dr Maria Alejandra Pinero de Plaza, PhD

**Affiliations:** 1 College of Nursing and Health Sciences, Flinders University, Adelaide, South Australia, Australia

I have thoroughly reviewed the amended version of the article and have no further comments. I commend the research team for their outstanding work and dedication to this research.

Is the rationale for developing the new method (or application) clearly explained?

Yes

Is the description of the method technically sound?

Yes

Are the conclusions about the method and its performance adequately supported by the findings presented in the article?

Yes

If any results are presented, are all the source data underlying the results available to ensure full reproducibility?

Yes

Are sufficient details provided to allow replication of the method development and its use by others?

Yes

Reviewer Expertise:

My research focuses on public health and improving healthcare services. I use different methods to find effective ways to promote healthy lifestyles and tackle health disparities. I also study how technology affects people's behaviour and how research findings can benefit society. By contributing to evaluation and implementation science, my goal is to turn research discoveries into practical solutions that can improve healthcare delivery, public health outcomes, and the development of strong health and care networks.

I confirm that I have read this submission and believe that I have an appropriate level of expertise to confirm that it is of an acceptable scientific standard.

# Reviewer report: Reviewer response for version 1

**DOI:** 10.5256/f1000research.154206.r243163

**Published:** 2024-02-29

**Authors:** Dr Maria Alejandra Pinero de Plaza, PhD

**Affiliations:** 1 College of Nursing and Health Sciences, Flinders University, Adelaide, South Australia, Australia

This work introduces SCOPE, a five-stage framework used to plan, design, and conduct research evaluations. The method has been successfully applied in various organisational settings globally and offers valuable insights for replication by other organisations to adopt similar evaluation practices. Four case studies demonstrate how SCOPE has been utilised to provide value-based research evaluations. The method is part of international efforts towards more meaningful and robust research evaluation practices. The case studies show that SCOPE can be adapted to different organisational settings and levels of detail. The framework's technical soundness is clearly explained in its systematic approach to planning, designing, and conducting research evaluations. The detailed process descriptions and outcomes of each case study further validate the framework's practical application. The approach is used to enhance research culture, develop assessment mechanisms, and design national research assessment exercises.

 I suggest enhancing the background section by including insights from seminal works and the latest innovative evaluation methodologies. This addition would provide a robust framing and foundation to introduce the SCOPE framework. Referencing seminal works such as "Evaluation: A Systematic Approach" and "Utilization-Focused Evaluation" can offer comprehensive perspectives to the reader on evaluation methods and stakeholder involvement while incorporating innovative methodologies like “Co-designing, measuring, and optimizing innovations and solutions within complex adaptive health systems,” also known as PROLIFERATE. These addendums align with responsible research assessment principles advocated by initiatives like DORA, the Leiden Manifesto and the Hong Kong Principles. Integrating these suggestions into the background would not only contextualise SCOPE's significance within the evolving research evaluation landscape but also demonstrate a comprehensive understanding of the field, thereby strengthening its contribution to promoting responsible research practices.

Is the rationale for developing the new method (or application) clearly explained?

Yes

Is the description of the method technically sound?

Yes

Are the conclusions about the method and its performance adequately supported by the findings presented in the article?

Yes

If any results are presented, are all the source data underlying the results available to ensure full reproducibility?

Yes

Are sufficient details provided to allow replication of the method development and its use by others?

Yes

Reviewer Expertise:

My research focuses on public health and improving healthcare services. I use different methods to find effective ways to promote healthy lifestyles and tackle health disparities. I also study how technology affects people's behaviour and how research findings can benefit society. By contributing to evaluation and implementation science, my goal is to turn research discoveries into practical solutions that can improve healthcare delivery, public health outcomes, and the development of strong health and care networks.

I confirm that I have read this submission and believe that I have an appropriate level of expertise to confirm that it is of an acceptable scientific standard, however I have significant reservations, as outlined above.

## References

[1] : Co-designing, measuring, and optimizing innovations and solutions within complex adaptive health systems. *Front Health Serv* .2023;3: 10.3389/frhs.2023.1154614 1154614 10.3389/frhs.2023.1154614 37063372 PMC10103186

# Reviewer report: Reviewer response for version 1

**DOI:** 10.5256/f1000research.154206.r218194

**Published:** 2023-12-19

**Authors:** Sven E. Hug

**Affiliations:** 1 Department of Psychology, University of Zurich, Zürich, Switzerland

The SCOPE framework is a significant milestone in the evolution of the field of research evaluation, as it is the first framework – apart from some societal impact frameworks – that is comprehensive in scope and introduces a procedure for designing evaluations. It is likely that the framework will pave the way for the professionalisation of research evaluation and spark more work on research evaluation frameworks, approaches, and methods.

 The three principles and the five steps presented in sections 5 and 6 are sound, consistent, reasonable, and comprehensible. The use cases presented in section 7 are clearly structured and described; they demonstrate the wide applicability of the framework. Even though the three principles and five steps could be specified, modified, and supplemented in many respects, I make no suggestions in this regard for two reasons. First, the framework in its current form is already productive and useful in informing research evaluation practice. Practical experience with the framework will undoubtedly trigger a further development of the framework. Second, the framework can serve as a benchmark and starting point for scholars and practitioners to either supplement the SCOPE framework or develop their own research evaluation frameworks. In my view, it is therefore not necessary nor desirable to change the framework presented in this paper or the accompanying 19-page guide at this time.

 While the framework itself is sound, there are three issues with how it is presented in the paper that require attention.

 (1) The paper promises too much.

 The title promises that the framework will “implement the ideals of responsible research assessment (RRA)”. The paper, however, does not provide a definition of RRA (section 2), only briefly discusses some RRA values and principles (section 3), and describes the relationship between RRA values/principles and the SCOPE framework only briefly and broadly (section 4, table 1).

 A minimum solution would be to remove the definite article from the title (implementing the ideals > implementing ideals) and to indicate that there are more RRA values/principles and that RRA values/principles are emerging and therefore vague. A maximum solution would be to provide a definition of RRA (or: a discussion of RRA definitions), discuss and list RRA values/principles comprehensively (or at least in more detail), and describe the relationship between RRA values/principles and the SCOPE framework in detail.

 (2) Responsible research assessment is not sufficiently described and discussed.

 Section 2 focuses on RRA. The last paragraph suggests that the negative consequences of (all) evaluation methods – and the awareness of them – are a recent phenomenon. Such concerns, however, are almost as old as evaluative bibliometrics and modern peer review. These concerns are therefore not sufficient to explain the surge of reform proposals and initiatives. Moreover, the section does not discuss definitions of RRA or reflect on RRA. This is surprising as one of the authors of this paper has written a blog on this very topic (https://www.leidenmadtrics.nl/articles/navigating-responsible-research-assessment-guidelines).

 Section 2 could benefit from a more thorough discussion of RRA, including, for example, Curry et al. (2020), Rushforth and Hammarfelt (2023), Rushforth and de Rijcke (2023), Peruginelli and Pölönen (2023) and Gauffriau ( https://www.leidenmadtrics.nl/articles/navigating-responsible-research-assessment-guidelines).

 (3) The section “Discussion and conclusions” is not very critical.

 Section 8 contains a lot of praise for the framework but does not really point out weaknesses and limitations. For example, the authors state that the simplicity of the framework is both a strength and a weakness. In what respect is it a strength and a weakness? What does this imply for practice and the further development of the framework? Moreover, section 8 does not discuss future research.

 I therefore encourage the authors to write a more balanced discussion of the strengths, weaknesses, and limitations of the framework, and to delineate future research.

 Curiosity question from the reviewer, not to be addressed in the paper:

 I count eight examples of use cases in the paper and ten on the webpage, four of which are referenced in the paper. This makes 14 use cases in total. The paper says on p. 10 that “the SCOPE framework is in wide usage by a range of organisations globally.” Does this imply that SCOPE is used in more than the 14 cases?

Is the rationale for developing the new method (or application) clearly explained?

Yes

Is the description of the method technically sound?

Yes

Are the conclusions about the method and its performance adequately supported by the findings presented in the article?

Partly

If any results are presented, are all the source data underlying the results available to ensure full reproducibility?

No source data required

Are sufficient details provided to allow replication of the method development and its use by others?

Yes

Reviewer Expertise:

Research evaluation, bibliometrics, peer review

I confirm that I have read this submission and believe that I have an appropriate level of expertise to confirm that it is of an acceptable scientific standard, however I have significant reservations, as outlined above.

## References

[1] : The changing role of funders in responsible research assessment: Progress, obstacles and the way ahead dust. *Research on Research Institute* .2020; 10.6084/m9.figshare.13227914.v1 10.6084/m9.figshare.13227914.v1

[2] : The legal foundation of responsible research assessment: An overview on European Union and Italy dust. *Research evaluation* .2023;00 issue: https://doi.org/10.1093/reseval/rvad035 10.1093/reseval/rvad035

[3] : Practicing responsible research assessment: Qualitative study of faculty hiring, promotion, and tenure assessments in the United States. *SocArXiv* .2023;0(0) : https://doi.org/10.31235/osf.io/2d7ax 10.31235/osf.io/2d7ax

[4] : The rise of responsible metrics as a professional reform movement: A collective action frames account. dust. *Quantitative Science Studies* .2023;1(37) : https://doi.org/10.1162/qss_a_00280 10.1162/qss_a_00280
